# FT3/FT4 ratio predicts non-alcoholic fatty liver disease independent of metabolic parameters in patients with euthyroidism and hypothyroidism

**DOI:** 10.6061/clinics/2016(04)08

**Published:** 2016-04

**Authors:** Fatma Yahyaoğlu Gökmen, Süleyman Ahbab, Hayriye Esra Ataoğlu, Betül Çavuşoğlu Türker, Faik Çetin, Fatih Türker, Rabia Yahyaoğlu Mamaç, Mustafa Yenigün

**Affiliations:** Haseki Training and Research Hospital, Internal Medicine Clinic, Istanbul, Turkey

**Keywords:** FT3/FT4 ratio, Insulin resistance, Non-alcoholic fatty liver disease, Hypothyroidism, Euthyroidism

## Abstract

**OBJECTIVE:**

This study was performed to evaluate the effects of metabolic parameters and thyroid dysfunction on the development of non-alcoholic fatty liver disease (NAFLD).

**METHODS:**

The current study evaluated a total of 115 patients, 75 female and 40 male. Physical examination and anthropometric measurements were applied to all participants. Hypothyroidism was considered at a thyroid stimulating hormone level ≥ 4.1 mIU/L. Patients with euthyroidism and patients with hypothyroidism were compared. Abdominal ultrasonography was used to diagnose non-alcoholic fatty liver disease. The participants were further compared with regard to the presence of non-alcoholic fatty liver disease. Logistic regression modeling was performed to identify the relationship between non-alcoholic fatty liver disease and independent variables, such as metabolic parameters and insulin resistance.

**RESULTS:**

Non-alcoholic fatty liver disease was identified in 69 patients. The mean waist circumference, body mass index, fasting plasma insulin, HOMA-IR (*p*<0.001) and FT_3_/FT_4_ ratio (*p*=0.01) values were significantly higher in the patients with NAFLD compared to those without it. Multivariate regression analysis revealed that FT_3_/FT_4_ ratio, waist circumference and insulin resistance were independent risk factors for non-alcoholic fatty liver disease.

**CONCLUSION:**

Insulin resistance, enlarged waist circumference, elevated body mass index, higher FT_3_/FT_4_ ratio and hypertriglyceridemia are independent risk factors for NADLF, whereas hypothyroidism is not directly related to the condition.

## INTRODUCTION

Non-alcoholic fatty liver disease (NAFLD), a pathological spectrum of chronic liver diseases ranging from simple steatosis to non-alcoholic steatohepatitis (NASH) with inflammation, has a high risk for progression to cirrhosis 1,2-3. NAFLD is a growing diagnosis and the most commonly encountered liver pathology in clinical practice 4,5. NAFLD is commonly asymptomatic and discovered incidentally. The diagnosis of NAFLD is based on exclusion criteria, such as alcohol consumption (more than 20 g/day), autoimmune liver disease, viral hepatitis infection, hemochromatosis, Wilson's disease, and drug consumption. All of these must be excluded before considering NAFLD 6. The prevalence of NAFLD is associated with abdominal obesity, diabetes mellitus and other metabolic risk factors 7, 8. NAFLD is a strong determinant for the development of metabolic syndrome, which has potentially relevant clinical implications with regard to diagnosis, prevention and treatment 9,10. Moreover, metabolic syndrome, insulin resistance, diabetes, obesity and mixed hyperlipidemia are major metabolic risk factors for NAFLD 11. Because of the hyperinsulinism, pro-thrombotic potential, and subclinical inflammation associated with NAFLD, patients with this condition are at increased risk for cardiovascular mortality 12. In addition, the correction of insulin resistance may not be sufficient to successfully treat NASH in the majority of patients, conflicting with previous studies on NAFLD pathogenesis 13.

The thyroid gland is significantly involved in lipid and carbohydrate metabolism, regulation of body weight and adipogenesis 14. Recent studies have suggested that thyroid dysfunction may play a role in NAFLD. Subclinical hypothyroidism is associated with metabolic syndrome, cardiovascular mortality, and disturbance of lipid metabolism 15, 16. Thyroid dysfunctions in the form of overt or subclinical hypothyroidism are prevalent among patients with NAFLD/NASH 17.

NAFLD is a risk factor for the development of type 2 diabetes, which is, in turn, a major contributor to progressive liver disease 18. In contrast, chronic infections, such as that caused by hepatitis C virus, have an association with the development of NAFLD, insulin resistance and metabolic parameters 19. The identification of risk factors is essential for preventing NAFLD. Therefore, in the current study, we evaluated the effects of metabolic parameters and thyroid dysfunction on the development of NAFLD.

## METHODS

### Participants

The current study evaluated 115 individuals, 75 female and 40 male, who were admitted to the Haseki Training and Research Hospital's outpatient clinic for routine care from July 2014 through January 2015. Anthropometric measurements were taken, and thyroid function tests were performed. Hypothyroidism was described according to Clinical Practice Guidelines for Hypothyroidism in Adults: Cosponsored by the American Association of Clinical Endocrinologists and the American Thyroid Association 20. Euthyroidism (ET) was described as a thyroid stimulating hormone (TSH) level of 0.5—4 mIU/L and no history of chronic disease. Hypothyroidism (HT) was described as a TSH level of ≥ 4.1 mIU/L. Patients meeting the following criteria were excluded: chronic liver and kidney disease, viral hepatitis, diabetes mellitus, undergoing corticosteroid treatment, malignancy, alcohol consumption greater than 20 g/d, and pregnancy. Informed consent was obtained from all participants. The study protocol was approved by the local ethics committee of Istanbul Haseki Training and Research Hospital.

### Measurements

All patients underwent physical examination. Blood pressure was measured using a mercury sphygmomanometer. Height (m), weight (kg), and waist circumference (WC) were also measured. WC was measured between the lowest rib and the crista iliaca superior. Body mass index (BMI) was calculated as weight (kg)/height (m)^2^. Plasma TSH, free T3 (FT3), free T4 (FT4), alanine aminotransferase (ALT), aspartate alanine aminotransferase (AST), gamma glutamyl transferase (GGT), alkaline phosphatase (ALP), glucose, insulin, total cholesterol, triglycerides, HDL and LDL cholesterol, uric acid and creatinine were measured after an 8-hour fast using an Abbot Architect Analyzer System (IL, USA). The homeostasis model assessment for insulin resistance (HOMA-IR) was calculated using the following formula: fasting blood glucose (mmol/l) × [insulin (mU/l)/22.5].

### Abdominal Ultrasonography

The presence of qualitative steatosis was determined using a standard 2D abdominal ultrasonography (USG). All participants underwent abdominal USG (Philips Active Array, 2D-Clearvue 550 device). NAFLD was characterized by the presence of hepatic brightness, hepatorenal echo contrast, deep attenuation and vascular blurring on USG 21.

### Statistical Analysis

Numeric values were expressed as the mean ± standard deviation. Statistical analysis was performed using SPSS 16.0 for Windows. The Kolmogorov-Smirnov Z test was used to determine the distributions of variables. Regular variances were assessed using a t test, and irregular variables were assessed using the Mann-Whitney U test. Logistic regression modeling was performed to assess independent risk factors of NAFLD. A *p* value <0.05 was considered statistically significant.

## RESULTS

In total, 115 participants were enrolled in this study: 54 presented with HT (F/M, 39/15) and 61 presented with ET (F/M, 36/25). The anthropometric and metabolic parameters of the patients with ET and HT were compared and are presented in Table 1. No significant differences were found in gender, age, mean BMI, systolic BP, diastolic BP, ALT, AST, ALP, GGT, total cholesterol, triglycerides, LDL cholesterol, HDL cholesterol, uric acid, fasting glucose, fasting insulin, HOMA-IR, NAFLD or ferritin in the subjects with ET (30.28 ± 5.19) versus those with HT. The mean FT_3_/FT_4_ ratio of the patients with HT was higher than that of the subjects with ET, at 4.61 ± 1.38 versus 3.63 ± 0.68, respectively (*p*<0.001). There was no difference in NAFLD status between the patients with ET and those with HT. NAFLD was identified in 69 of total 115 subjects: 33 patients with ET and 36 patients with HT.

The participants were compared according to the presence of NAFLD, and the parameters of the comparison are presented in Table 2. The mean WC, BMI, systolic and diastolic blood pressure values were statistically higher in the patients with NAFLD than those without the condition (*p*<0.001, <0.001, 0.049 and 0.003, respectively). Additionally, the patients with NAFLD had significantly higher triglyceride levels (164.96 ± 77.27 mg/dl) than those without NAFLD (112.61 ± 89.80 mg/dl) (*p*=0.001). The patients with NAFLD also had significantly higher uric acid, fasting insulin, HOMA-IR and FT_3_/FT_4_ ratios.

The subjects with ET or HT in this study were also compared according to the presence or absence of NAFLD, as shown in Table 3. The patients with ET and NAFLD had higher WC (*p*=0.001), total cholesterol (*p*=0.042), triglycerides (*p*<0.001), fasting insulin (*p*<0.001) and HOMA-IR (*p*=0.001) levels compared to the subjects with ET without NAFLD. While the FT_4_ levels in the patients with ET and NAFLD were lower than those in the patients with ET without NAFLD, the patients with ET and NAFLD had increased FT_3_/FT_4_ ratios, as well as uric acid, fasting insulin and HOMA-IR levels, compared to the patients with ET without NAFLD (*p*=0.01).

The patients with HT and NAFLD had lower FT_4_ levels compared to the patients with HT without NAFLD (Table 3). Additionally, the patients with HT and NAFLD had higher WC, total cholesterol, triglycerides, fasting insulin and HOMA-IR levels than the patients with HT without NAFLD.

Logistic regression analysis was performed to delineate the nature of the relationships that exist between NAFLD, metabolic parameters and insulin resistance as independent variables (Table 4). WC (OR: 1.087, *p*=0.01), HOMA-IR (OR: 2.978, *p*=0.005), and FT_3_/FT_4_ ratio (OR: 1.834, *p*=0.02) were independent risk factors for NAFLD in all study participants. Additional regression analysis was performed to evaluate HT patients with NAFLD with respect to metabolic parameters (Table 5). WC (OR: 1.189, *p*=0.02), triglycerides (OR: 1.031, *p*=0.04), uric acid (OR: 0.318, *p*=0.03), HOMA-IR (OR: 8.042, *p*=0.02) and FT_3_/FT_4_ ratio (OR: 3.540, *p*=0.01) were independent risk factors for NAFLD in patients with HT.

## DISCUSSION

NAFLD is a burgeoning health problem and is currently recognized as the most common metabolic liver disease. Insulin resistance and obesity contribute to the development of NAFLD, which has become the most prevalent liver disease worldwide, affecting one-third of the global adult population 22,23. NAFLD can lead to NASH and/or hepatocellular cancer 24.

It has been suggested that a relationship exists between NAFLD and thyroid dysfunction 25. Despite the precise physiological mechanism underlying the development of NAFLD, the relationship between NAFLD, hypothyroidism and metabolic syndrome remains unclear. Because of the importance of thyroid hormones in lipid metabolism 26, HT may result in hyperlipidemia, thereby initiating the development of NAFLD. Several studies have indicated that hypothyroidism is a risk factor for NAFLD and can result in metabolic syndrome 16,27,28. FT_3_/FT_4_ ratio can be considered an indicator of peripheral deiodinase activity. Bilgin and Pirgon 29 suggested that augmented conversion from FT4 to FT3 due to increased deiodinase activity is a compensatory mechanism for fat accumulation to improve energy expenditure. FT_3_/FT_4_ ratio positively correlates with HOMA-IR in patients with NAFLD 18. Moreover, positive associations have been reported between FT3/FT4 ratio and both waist circumference and BMI in patients with obesity 30. Ittermann and Haring 31 reported that low FT_4_ levels, but not low TSH and FT_3_ levels, are associated with hepatic steatosis. In the present study, the mean BMI values in patients with ET and patients with HT were similar; however, the patients with HT had significantly higher FT_3_/FT_4_ ratios (*p*<0.001). The patients with NAFLD had significantly elevated BMI, WC, HOMA-IR values and FT_3_/FT_4_ ratios; their FT_4_ levels were low, leading to increased FT_3_/FT_4_ ratios, but their TSH levels were unaffected. The results of this study suggest that elevated FT_3_/FT_4_ ratio is an independent risk factor for NAFLD.

Patients with HT have elevated triglyceride and LDL cholesterol levels due to decreased plasma lipoprotein lipase activity. Hyperlipidemia associated with fatty accumulation in the liver and cellular oxidative stress is one potential mechanism underlying the development of NAFLD 32,33. The results of the present study support this relationship, as TC (*p*=0.002), LDL cholesterol (*p*=0.001), triglyceride (*p*=0.008), and uric acid (*p*=0.006) levels were significantly higher and HDL cholesterol levels lower (*p*=0.022) in the patients with NAFLD.

The prevalence of NAFLD did not significantly differ between the ET (n:36, 59%) and HT (n:33, 64%) groups. Mazo and Lima 34 previously reported that no association exists between HT, hepatosteatosis and NASH. In addition, Eshraghian and Dabbaghmanesh 35 reported that no association exists between autoimmune thyroid disorder and elevated anti-thyroid peroxidase levels, anti-tiroglobulin levels and NAFLD. Furthermore, NAFLD was not correlated with thyroid dysfunction in the current study, as the included patients with ET and HT did not show significant differences in NAFLD prevalence, insulin resistance, abdominal obesity or BMI. The mean BMI of the patients with ET and HT was above 30, and abdominal obesity was considered to be more important to the development of NAFLD than HT. WC, FT_3_/FT_4_ ratio, triglyceride level and serum uric acid level were independent risk factors for NAFLD in the patients with HT in our study. Abdominal obesity is a substantial component of metabolic syndrome and increases the risk for cardiovascular events. Visceral fat can be considered an important predictive factor for NAFLD 36.

The mean WC (*p*<0.001), BMI (*p*<0.001), systolic and diastolic blood pressure (*p*=0.049 and *p*=0.003) values were higher in the patients with NAFLD in the present study. Fatty liver disease has been associated with anthropometric findings. Moreover, abdominal obesity and increased WC have been associated with NAFLD 37. Huang and Beilin 38 demonstrated that systolic and diastolic blood pressure are elevated in patients with NAFLD compared to controls. Concordant with the referenced results, in the current study, AST, ALT and GGT were increased in patients with NAFLD. Chung and Kim 25 reported that both the prevalence of NAFLD and ALT levels were higher in patients with HT. Serum ALT level is a surrogate marker for NAFLD in the absence of other causes of liver disease 17. In the current study, fasting insulin and HOMA-IR values were elevated in patients with NAFLD. Furthermore, insulin resistance and fasting insulin level formed a strong relationship with NAFLD, independent of HT. Additionally, it has been reported that hyperinsulinemia and HT can separately result in the development of NAFLD 39,40.

In the current study, abdominal USG was applied to diagnose NAFLD via the qualitative detection of steatosis. Abdominal USG detects changes in fatty accumulation in the liver of as low as ≥20% and closely mirrors coronary and carotid atherosclerosis burden. In contrast, semi-quantitative USG indices (to exclude NASH) and sonoelastography (to quantify fibrosis) help predict liver histology and can be used to help select patients to submit to liver biopsy 41. According to the above, semi-quantitative steatosis indices must be further investigated.

In conclusion, FT_3_/FT_4_ ratio, HOMA-IR and WC are risk factors for the development of NAFLD. FT_3_/FT_4_ ratio is a predictor of NAFLD independent of insulin resistance both in patients with ET and in patients with HT. Elevated serum triglyceride and uric acid levels are independent risk factors for NAFLD in patients with HT.

## AUTHOR CONTRIBUTIONS

Gökmen FY and Ahbab S participated in the study design, study coordination and drafting of the manuscript. Ataoğlu HE participated in statistic analysis and helped in drafting the manuscript. Türker BÇ, Çetin F, Türker F and Mamaç RY participated in data collection. Yenigün M participated in study design and coordination.

## Figures and Tables

**Table 1:** Parameters compared between patients with euthyroidism and those with hypothyroidism.

Parameters	Patients with ET (n:61)	Patients with HT (n:54)	*p* value
**Gender, male %**	41%	27.8%	0.138
**Age**	48.44 ± 13.19	47.98 ± 11.87	0.845
**WC (cm)**	94.34 ± 11.00	92.11 ± 12.59	0.312
**BMI**	30.28 ± 5.19	30.05 ± 6.54	0.831
**Systolic BP (mm/Hg)**	123.20 ± 18.14	118.24 ± 18.14	0.162
**Diastolic BP (mm/Hg)**	77.05 ± 11.45	73.61 ± 11.30	0.109
**TSH (mIU/L)**	1.63 ± 0.91	22.48 ± 15.91	**<0.001**
**FT3 (pg/ml)**	0.86 ± 0.11	0.62 ± 0.17	**<0.001**
**FT4 (ng/dl)**	3.07 ± 0.44	2.66 ± 0.37	**<0.001**
**FT3**/FT4</emph> ratio</emph>	3.63 ± 0.68	4.61 ± 1.48	**<0.001**
**ALT (mg/dl)**	24.64 ± 15.67	21.76 ± 11.53	0.261
**AST (mg/dl)**	24.98 ± 8.14	24.37 ± 9.87	0.719
**ALP (mg/dl)**	79.96 ± 24.51	86.11 ± 28.26	0.22
**GGT (mg/dl)**	28.96 ± 20.42	28.40 ± 25.46	0.896
**Total cholesterol (mg/dl)**	203.77 ± 48.74	208.19 ± 43.87	0.613
**Triglycerides (mg/dl)**	131.11 ± 88.01	158.59 ± 82.20	0.088
**LDL cholesterol (mg/dl)**	127.92 ± 39.25	126.83 ± 36.89	0.879
**HDL cholesterol (mg/dl)**	51.04 ± 9.97	49.66 ± 11.05	0.484
**Uric acid (mg/dl)**	5.10 ± 1.40	4.96 ± 1.54	0.705
**Fasting glucose (mg/dl)**	99.69 ± 21.04	103.41 ± 39.09	0.52
**Fasting insulin (mIU/ml)**	8.83 ± 5.23	8.59 ± 4.87	0.804
**HOMA-IR**	2.15 ± 1.32	2.11 ± 1.26	0.894
**NAFLD (n - %)**	36 - 59%	33 - 64%	0.819
**Ferritin (ng/ml)**	61.79 ± 91.20	29.12 ± 18.77	0.099

Euthyroidism, hypothyroidism n: number of patients. WC: waist circumference. BMI: body mass index. BP: blood pressure. ALT: alanine aminotransferase. AST: aspartate aminotransferase. ALP: alkaline phosphatase. GGT: gamma glutamyl transferase. HOMA-IR: homeostasis model assessment for insulin resistance. NAFLD: non-alcoholic fatty liver disease.

**Table 2:** Comparison of study parameters between patients with and without non-alcoholic fatty liver disease.

Parameters	Without NAFLD (n:46)	With NAFLD (n:69)	*p* value
**Gender, male %**	28.3%	39.1%	0.231
**Age**	45.76 ± 12.30	49.87 ± 12.51	0.085
**WC (cm)**	86.20 ± 12.73	98.03 ± 8.27	**<0.001**
**BMI**	27.11 ± 5.29	32.21 ± 5.30	**<0.001**
**Systolic BP (mm/Hg)**	116.63 ± 17.80	123.70 ± 19.24	**0.049**
**Diastolic BP (mm/Hg)**	71.63 ± 9.95	77.97 ± 11.77	**0.003**
**TSH (mIU/L)**	9.51 ± 10.33	13.81 ± 20.30	0.138
**FT3** (pg/ml)</emph>	2.91 ± 0.34	2.85 ± 0.52	0.479
**FT4** (ng/dl)</emph>	0.80 ± 0.16	0.72 ± 0.20	**0.025**
**FT3/FT4 ratio**	3.78 ± 0.82	4.32 ± 1.42	**0.015**
**ALT (mg/dl)**	18.02 ± 9.34	26.80 ± 15.32	**<0.001**
**AST (mg/dl)**	21.62 ± 5.87	26.74 ± 10.05	**0.001**
**ALP (mg/dl)**	79.46 ± 24.38	84.98 ± 27.54	0.28
**GGT (mg/dl)**	21.97 ± 18.29	33.15 ± 24.43	**0.01**
**Total cholesterol (mg/dl)**	189.89 ± 42.22	216.48 ± 46.23	**0.002**
**Triglycerides (mg/dl)**	112.61 ± 89.80	164.96 ± 77.27	**0.001**
**LDL cholesterol (mg/dl)**	115.93 ± 33.98	135.06 ± 38.83	**0.008**
**HDL cholesterol (mg/dl)**	53.22 ± 11.41	48.50 ± 9.41	**0.022**
**Uric acid (mg/dl)**	4.58 ± 1.46	5.36 ± 1.40	**0.006**
**Fasting glucose (mg/dl)**	102.17 ± 46.61	100.94 ± 12.20	0.862
**Fasting insulin (mIU/ml)**	5.72 ± 2.30	10.58 ± 5.38	**<0.001**
**HOMA-IR**	1.37 ± 0.60	2.62 ± 1.36	**<0.001**
**Ferritin (ng/ml)**	23.83 ± 12.62	54.80 ± 79.08	0.187

Non-alcoholic fatty liver disease. WC: waist circumference. BMI: body mass index. BP: blood pressure. ALT: alanine aminotransferase. AST: aspartate aminotransferase. ALP: alkaline phosphatase. GGT: gamma glutamyl transferase. HOMA-IR: homeostasis model assessment for insulin resistance.

**Table 3:** Comparison of parameters between patients with ET or HT according to presence or absence of non-alcoholic fatty liver disease.

	Parameters	with NAFLD	without NAFLD	*p* value
	**N**	36	25	-
	**Age**	49.78 ± 12.77	46.52 ± 13.80	0.347
*Patients*	**FT3** (pg/ml)</emph>	3.09 ± 0.52	3.04 ± 0.29	0.645
*with*	**FT4** (ng/dl)</emph>	0.84 ± 0.11	0.90 ± 0.10	**0.046**
*euthyroidism*	**FT3/FT4 ratio**	3.78 ± 0.78	3.42 ± 0.47	**0.01**
	**TSH (mIU/L)**	1.71 ± 0.96	1.50 ± 0.83	0.381
	**WC (cm)**	98.86 ± 7.69	87.84 ± 11.90	**<0.001**
	**Total cholesterol (mg/dl)**	215.25 ± 51.83	187.24 ± 39.20	**0.026**
	**LDL cholesterol (mg/dl)**	137.56 ± 41.72	114.04 ± 31.18	**0.02**
	**HDL cholesterol (mg/dl)**	49.06 ± 8.21	53.88 ± 11.65	0.082
	**Triglycerides (mg/dl)**	143.72 ± 66.64	112.96 ± 110.93	0.18
	**Glucose (mg/dl)**	101.97 ± 11.69	96.40 ± 29.79	0.313
	**Uric acid (mg/dl)**	5.50 ± 1.42	4.48 ± 1.15	**0.006**
	**Fasting insulin (mIU/ml)**	10.53 ± 5.82	6.05 ± 2.14	**<0.001**
	**HOMA-IR**	2.62 ± 1.43	1.39 ± 0.53	**<0.001**
	**N**	33	21	-
	**Age**	49.97 ± 12.42	44.86 ± 10.48	0.124
***Patients***	**FT3** (pg/ml)</emph>	27.01 ± 23.02	19.04 ± 79	0.075
***with***	**FT4** (ng/dl)</emph>	2.59 ± 0.37	2.76 ± 0.35	0.103
***hypothyroidism***	**FT3/FT4** ratio</emph>	0.59 ± 0.19	0.68 ± 0.13	**0.036**
	**TSH (mIU/L)**	4.89 ± 1.72	4.20 ± 0.94	0.27
	**WC (cm)**	97.12 ± 8.89	84.24 ± 13.67	**0.001**
	**Total cholesterol (mg/dl)**	217.82 ± 40.00	193.05 ± 46.34	**0.042**
	**LDL cholesterol (mg/dl)**	132.33 ± 35.85	118.19 ± 37.69	0.172
	**HDL cholesterol (mg/dl)**	47.89 ± 10.64	52.43 ± 11.34	0.14
	**Triglycerides (mg/dl)**	188.12 ± 82.28	112.19 ± 58.08	**<0.001**
	**Glucose (mg/dl)**	109.05 ± 61.09	99.82 ± 12.81	0.502
	**Uric acid (mg/dl)**	5.19 ± 1.38	4.70 ± 1.76	0.259
	**Fasting insulin (mIU/ml)**	10.64 ± 4.94	5.38 ± 2.44	**<0.001**
	**HOMA-IR**	2.61 ± 1.30	1.34 ± 0.67	**<0.001**

NAFLD: non-alcoholic fatty liver disease. FT3: free T3. FT4: free T4. TSH: thyroid stimulating hormone. WC: waist circumference. HOMA-IR: homeostasis model assessment for insulin resistance.

**Table 4:** Logistic regression analysis of the association between non-alcoholic fatty liver disease and metabolic variables in all participants.

Variables	*p* value	OR	95% CI
WC	**0.01**	1.087	1.018 - 1.061
Triglycerides	0.12	1.010	0.997 - 1.031
Total cholesterol	0.21	1.009	0.995 - 0.921
Uric acid	0.79	1.056	0.706 - 51.283
HOMA-IR	**0.005**	2.978	1.397 - 9.575
FT_3_/FT_4_ ratio	**0.02**	1.834	1.089 - 3.569

WC: waist circumference. HOMA-IR: homeostasis model assessment for insulin resistance. OR: odds ratio. CI: confidence interval.

**Table 5:** Logistic regression analysis of the association between non-alcoholic fatty liver disease and metabolic variables in patients with HT.

Variables	*p* value	OR	95% CI
WC	**0.02**	1.189	1.024 - 1.381
Triglycerides	**0.04**	1.031	1.001 - 1.061
Total cholesterol	0.78	1.004	0.977 - 1.031
Uric acid	**0.03**	0.318	0.11 - 0.921
HOMA-IR	**0.02**	8.042	1.261 - 51.283
FT_3_/FT_4_ ratio	**0.01**	3.540	1.309 - 9.575

WC: waist circumference. HOMA-IR: homeostasis model assessment for insulin resistance. OR: odds ratio. CI: confidence interval.
